# Decrease of disease‐related metabolites upon fasting in a hemizygous knock‐in mouse model (*Mut*‐ko/ki) of methylmalonic aciduria

**DOI:** 10.1002/jmd2.12182

**Published:** 2020-11-08

**Authors:** Marie Lucienne, Déborah Mathis, Nathan Perkins, Ralph Fingerhut, Matthias R. Baumgartner, D. Sean Froese

**Affiliations:** ^1^ Division of Metabolism and Children's Research Center University Children's Hospital Zurich Zurich Switzerland; ^2^ radiz – Rare Disease Initiative Zurich, Clinical Research Priority Program for Rare Diseases University of Zurich Zurich Switzerland; ^3^ Zurich Center for Integrative Human Physiology University of Zurich Zurich Switzerland; ^4^ Division of Clinical Chemistry and Biochemistry University Children's Hospital Zurich Zurich Switzerland; ^5^ Swiss Newborn Screening Laboratory University Children's Hospital Zurich Zurich Switzerland

**Keywords:** catabolism, disease amelioration, fasting, Methylmalonic aciduria, methylmalonyl‐CoA mutase, mouse model

## Abstract

Methylmalonyl‐CoA mutase (MMUT) is part of the propionyl‐CoA catabolic pathway, responsible for the breakdown of branched‐chain amino acids, odd‐chain fatty acids and the side‐chain of cholesterol. Patients with deficient activity of MMUT suffer from isolated methylmalonic aciduria (MMAuria), frequently presenting in the newborn period with failure to thrive and metabolic crisis. Even well managed patients remain at risk for metabolic crises, of which one known trigger is acute illness, which may lead to poor feeding and vomiting, putting the patient in a catabolic state. This situation is believed to result in increased breakdown of propionyl‐CoA catabolic pathway precursors, producing massively elevated levels of disease related metabolites, including methylmalonic acid and propionylcarnitine. Here, we used fasting of a hemizygous mouse model (*Mut*‐ko/ki) of MMUT deficiency to study the role of induced catabolism on metabolite production. Although mice lost weight and displayed markers consistent with a catabolic state, contrary to expectation, we found strongly reduced levels of methylmalonic acid and propionylcarnitine in fasted conditions. Switching *Mut*‐ko/ki mice from a high‐protein diet to fasted conditions, or from a standard diet to a no‐protein diet, resulted in similar reductions of methylmalonic acid and propionylcarnitine levels. These results suggest, in our mouse model at least, induction of a catabolic state on its own may not be sufficient to trigger elevated metabolite levels.

**Synopsis**

In contrast to expectations in the patient condition, induced catabolism of a mouse model of methylmalonic aciduria results in reduced rather than elevated concentrations of disease related metabolites.

## 1 INTRODUCTION

Isolated methylmalonic aciduria (MMAuria) is an inherited disorder of propionate metabolism typically caused by dysfunction of the enzyme methylmalonyl‐CoA mutase (MMUT) or a defect in the transport or processing of its organometallic cofactor adenosylcobalamin. The MMUT enzyme catalyzes the reversible isomerization of l‐methylmalonyl‐CoA into succinyl‐CoA, an intermediate in the tricarboxylic acid cycle and an important step in the breakdown of some essential amino acids (valine, isoleucine, methionine and threonine), odd‐chain fatty acids, and the side‐chain of cholesterol. Biochemically, MMAuria is characterized by the accumulation of methylmalonyl‐CoA and propionyl‐CoA derivatives, such as methylmalonic acid (MMA) and propionylcarnitine (C3) in tissues and body fluids.^1^, ^2^ Patients with MMAuria frequently present in the newborn period with failure to thrive, lethargy, repeated vomiting, and life‐threatening metabolic crises. In case of crisis‐like episodes, patients show highly elevated disease‐related metabolites and are kept anabolic in order to meet their energy needs and avoid prolonged fasting.^3^, ^4^ The presumed mechanism leading to acute crisis upon prolonged fasting is an induction of protein or odd‐chain fatty acid catabolism, which increases the throughput of the propionate pathway and production of toxic metabolites.^3^, ^4^, ^5^, ^6^, ^7^ Treatment recommendations suggest ensuring adequate energy supply, mainly in the form of carbohydrates, during situations of impaired intake and increased energy demands, such as intercurrent infections.^3^, ^4^

Homozygous knock‐out of *Mmut* in mice (*Mut*‐ko/ko) has been shown to result in neonatal lethality.^8^, ^9^ We therefore previously developed a hemizygous mouse model of MMAuria combining a knock‐in (ki) allele with a knock‐out (ko) allele (*Mut*‐ko/ki) of the *Mmut* gene, whereby the ki allele was based on the p.Met700Lys patient missense mutation (p.Met698Lys in mouse).^10^ These mice show many of the biochemical and clinical symptoms identified in humans, including elevated methylmalonic acid and C3 in blood and tissues, failure to thrive, and an increased severity when placed on a protein‐rich diet.^11^ This led to long‐term dysfunction across many organ systems, including mild neurological and kidney dysfunction, degenerative morphological changes in the liver, cardiovascular, and hematological abnormalities. In order to investigate the role of fasting as an exacerbator of disease, we deprived female *Mut*‐ko/ki and littermate control (*Mut*‐ki/wt) mice of food for up to 48 hours following ad libitum feeding with either protein‐rich or regular chow. We expected the mice to have very high metabolite levels and to show signs of metabolic crisis. Unexpectedly, the mice tolerated this intervention, and biochemical markers of MMAuria in blood and urine of *Mut*‐ko/ki mice were strongly reduced after 24 or 48 hours. The mechanisms governing these changes remain unclear, but these findings caution that fasting itself may not be a driver of increased disease related metabolites.

## 2 MATERIALS AND METHODS

### 2.1 Ethics statement

All animal experiments were approved by the legal authorities (licenses: 048/2016, 171/19; Kantonales Veterinäramt Zürich, Switzerland) and performed according to the legal and ethical requirements. All institutional and national guidelines for the care and use of laboratory animals were followed.

### 2.2 Animal housing and conditions

To generate the experimental *Mut*‐ko/ki and the control *Mut*‐ki/wt female mice used in this study, *Mut*‐ko/wt females were crossed with *Mut*‐ki/ki males. Mice were bred and housed in single‐ventilated cages with a 12:12 hour light/dark cycle and an artificial light of approximately 40 Lux in the cage. The animals were kept under controlled humidity (45%‐55%) and temperature (21 ± 1°C) and housed in a barrier‐protected specific pathogen‐free unit. Mice had ad libitum access to sterilized drinking water. In normal ad libitum fed conditions, mice had access to pelleted and extruded regular mouse chow containing 18.5% of protein (maintenance diet 3436, Kliba Nafag, Switzerland) given from birth. Mice were co‐housed, two to five to a cage, as a mixture of mutants and/or controls. Before food withdrawal or before being switched to a new dietary condition, mice were exchanged to cages with fresh bedding. When specified, mice had access to mouse customized diet containing 0% or 51% of protein and whose composition was based on the reference diet U8978 version 22 (Safe, France). The composition of the 0% protein diet was: 52.7% corn starch, 14.2% maltodextrin, 15.4% saccharose, 8% soy oil, 5% cellulose, 3.5% mineral mixture, 1% vitamin mixture, and 0.25% choline bitartate. They had ad libitum access to this diet. The 51% protein diet was given from day 12 of life as previously described.^11^ In fasting conditions, mice were placed in a new cage with no food for 24 or 48 hours, water ad libitum. Mouse monitoring entailed regular weight measurements and clinical observation. Apart from weight loss, we did not notice any change in the condition or behavior of the mice, including after up to 48 hours of fasting and under a 0% protein diet.

### 2.3 Metabolite measurements

Blood was collected from tail vein upon incision with sterilized scissors, without restraining the animal. Thirty microliters of blood were placed directly on a filter card and whole blood concentration of MMA, acetylcarnitine and propionylcarnitine was determined on dried blood spots.

Acylcarnitines and amino acids were extracted using the Neomass AAAC Plus newborn screening kit from Labsystems Diagnostics OY (Helsinki, Finland). A 25‐μL aliquot of the supernatant fluid was transferred into a separate vial and prepared for analysis of MMA using the ClinMass MMA advanced kit from Recipe Chemicals + instruments GmbH (München, Germany). A UHPLC Nexera X2 coupled to an LCMS‐8060 Triple Quadrupole mass spectrometer with electrospray ionization from Shimdazu (Kyōto, Japan) were used for the quantitative analysis of acylcarnitines, amino acids and MMA. Labsolutions and Neonatal Solutions software were used for data acquisition and data analysis. Measurements were performed in duplicates and averages were calculated prior to data analysis. Using the FreeStyle Precision Neo and the FreeStyle Precision β‐Ketone test strips (Abbott), 0.6 μL of whole blood were used to measure 3‐hydroxybutyrate concentrations.

Plasma was prepared by pooling ~50 μL of whole blood collected from tail vein and/or vena cava in a microvette 500 μL lithium heparin orange EU code (Sarstedt), followed by centrifugation at 1500*g* for 10 minutes at +4°C. Prior to blood collection from vena cava, mice were anaesthetized with a sedative solution (xylazine 35 mg/kg, ketamine 200 mg/kg, in NaCl 0.9%).

For determination of MMA in plasma and urine samples, plasma and urine (~20 and ~70 μL, respectively) were diluted with water (factor 2 and 20 for plasma and urine of control mice, respectively; factor 21‐441 and 50′000 for plasma and urine of mutant mice, respectively), to which internal standard (D3‐MMA, Cambridge Isotope Laboratories) was added. The samples were vortexed, centrifuged at 16000*g* for 5 minutes, the supernatant transferred to HPLC vials, and 2 μL injected into an UltiMate 3000 Rapid Separation LC coupled to an AB Sciex 5500 TripleQuad mass spectrometer (Thermo Scientific) in MRM mode using a commercial kit (Recipe ClinMass advanced). Creatinine was determined by enzymatic assay on a routine analyser Alinity C (Abbott Laboratories).

Data was visualized and statistical analysis performed using GraphPad Prism v.8.4.

## 3 RESULTS

**FIGURE 1:**
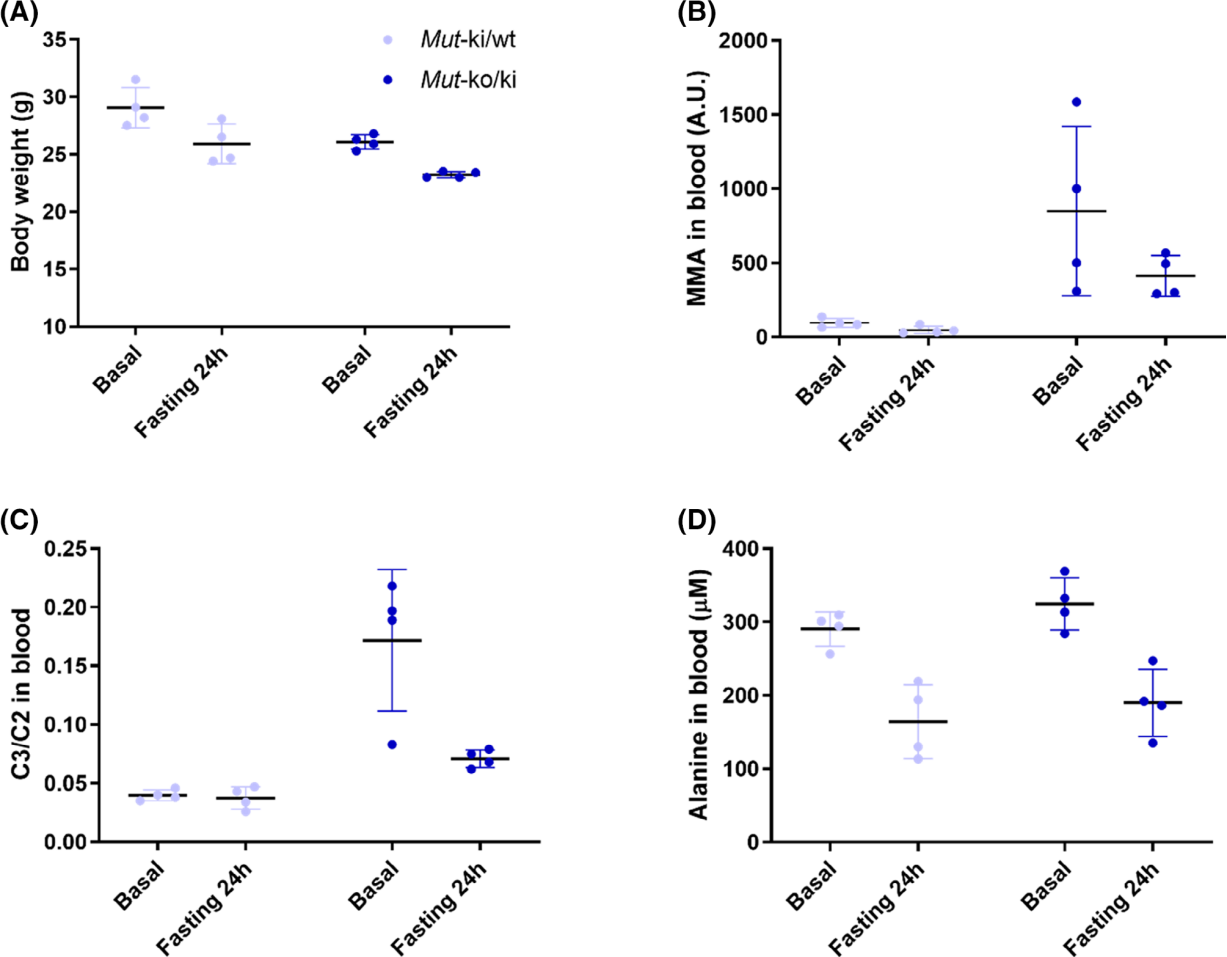
*Mut*‐ko/ki mice display a reduction of metabolite levels upon 24‐hour fasting. A, Changes in body weight, B, MMA, C, C3/C2 and, D, alanine in *Mut*‐ki/wt (n = 4) and *Mut*‐ko/ki (n = 4) female mice before and following 24 hours of fasting. MMA, C3/C2 and alanine were measured in dried blood spots. All changes between basal and fasting conditions for *Mut*‐ko/ki are significant by at least *P* < .05, except MMA in dried blood spots (*P* = .14). Changes between basal and fasting conditions for *Mut*‐ki/wt are significant for body weight and alanine. Paired t‐tests were used for these analyses. At the time of experiment mice were 3.5 months of age. Baseline levels were determined 1 day before the food withdrawal

In order to determine the response of *Mut*‐ko/ki mice to fasting, we investigated the metabolic profile of *Mut*‐ko/ki and *Mut‐*ki/wt mice in ad libitum fed conditions (regular chow) and following 24‐hour food withdrawal. Both sets of mice exhibited weight loss consistent with fasted conditions (Figure 1A). However, *Mut*‐ko/ki mice surprisingly appeared to show reduction in detectable MMA (Figure 1B), and did show reduced C3 normalized to acetylcarnitine (C2) (Figure 1C), following deprivation. This despite the fact that in both sets of mice circulating alanine concentrations were reduced (Figure 1D), consistent with the production of ketone bodies,^12^ and indicating catabolic conditions.

**FIGURE 2:**
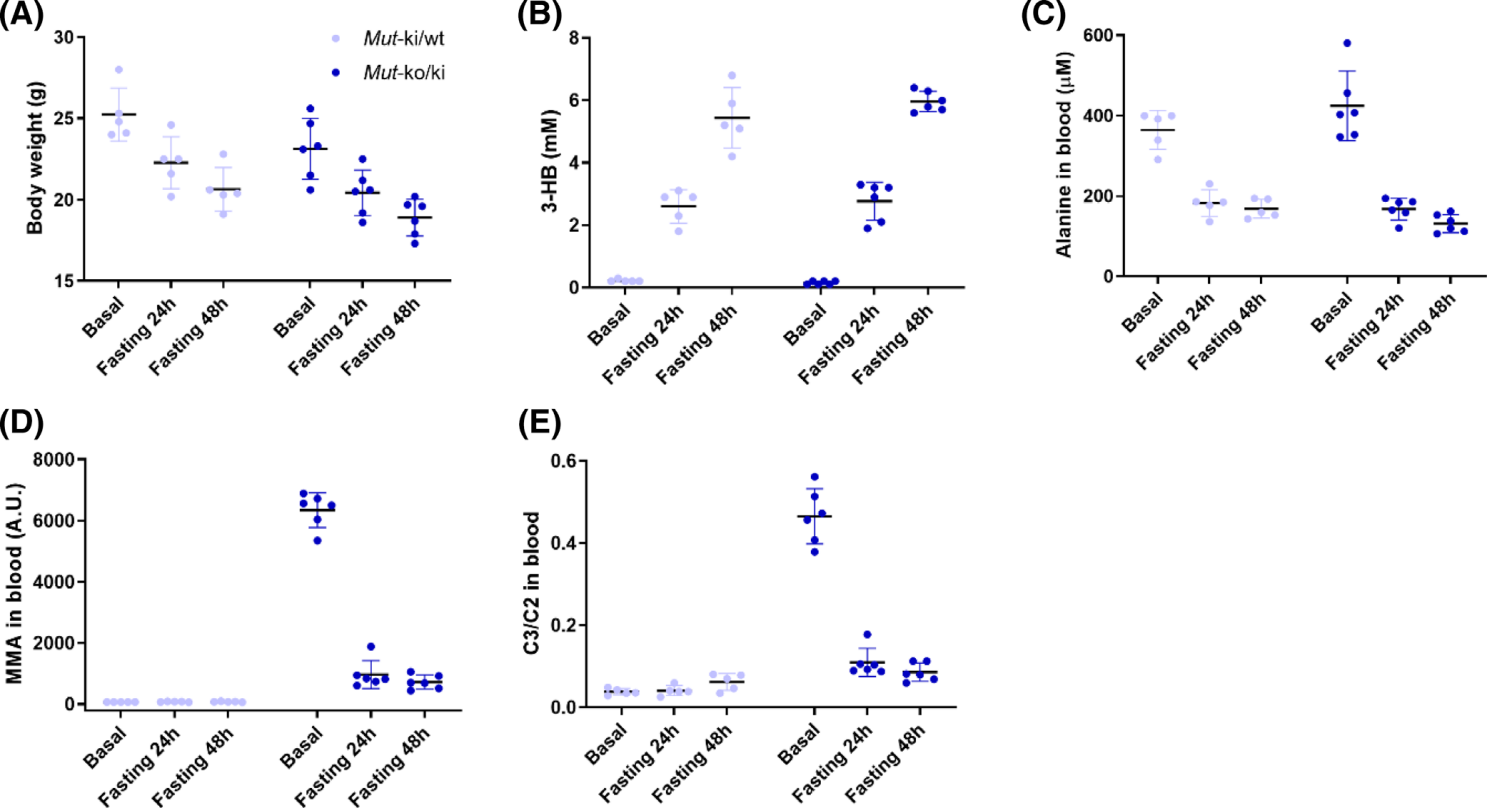
*Mut*‐ko/ki mice display a reduction of metabolite levels upon long‐term fasting. A, Changes in body weight, B, 3‐hydroxybutyrate (3‐HB) in whole blood, and C, alanine, D, MMA, and E, C3/C2 in dried blot spots were measured before, during (after 24 hours) and at the end of 48 hours of fasting in *Mut*‐ki/wt (n = 5) and *Mut*‐ko/ki (n = 6) females. In *Mut*‐ko/ki mice, MMA, C3/C2 and alanine levels decrease after 24 hours of fasting (*P* < .001) and stabilize, body weight decrease after 24 hours of fasting (*P* < .05), while 3‐HB increase at 24 hours (*P* < .001) and further increase at 48 hours (*P* < .001). Changes between basal and 24‐hour fasting conditions for *Mut*‐ki/wt are significant for body weight, 3‐hydroxybutyrate and alanine. Two‐way analysis of variance with Tukey's multiple comparison tests were used for these analyses. At the time of experiment mice were 4 months of age. Baseline levels were determined 17 days before the food withdrawal

In a further cohort of mice, we examined the response to fasting of up to 48 hours. Once again, throughout the course of fasting both sets of *Mut*‐ko/ki and *Mut‐*ki/wt mice lost weight (Figure 2A) and showed markers of catabolism, including elevated ketone bodies (3‐hydroxybutyrate, Figure 2B) and reduced alanine (Figure 2C) in the blood. Although baseline levels of C3/C2 and MMA in mutant animals were much more elevated than in the previous cohort (Figure 1B,C) and than in mutant mice fed a similar diet in former studies,^11^ *Mut*‐ko/ki mice had the same response to fasting as the previous cohort: a strong reduction of MMA (Figure 2D) and of C3/C2 (Figure 2E) in the blood.

**FIGURE 3:**
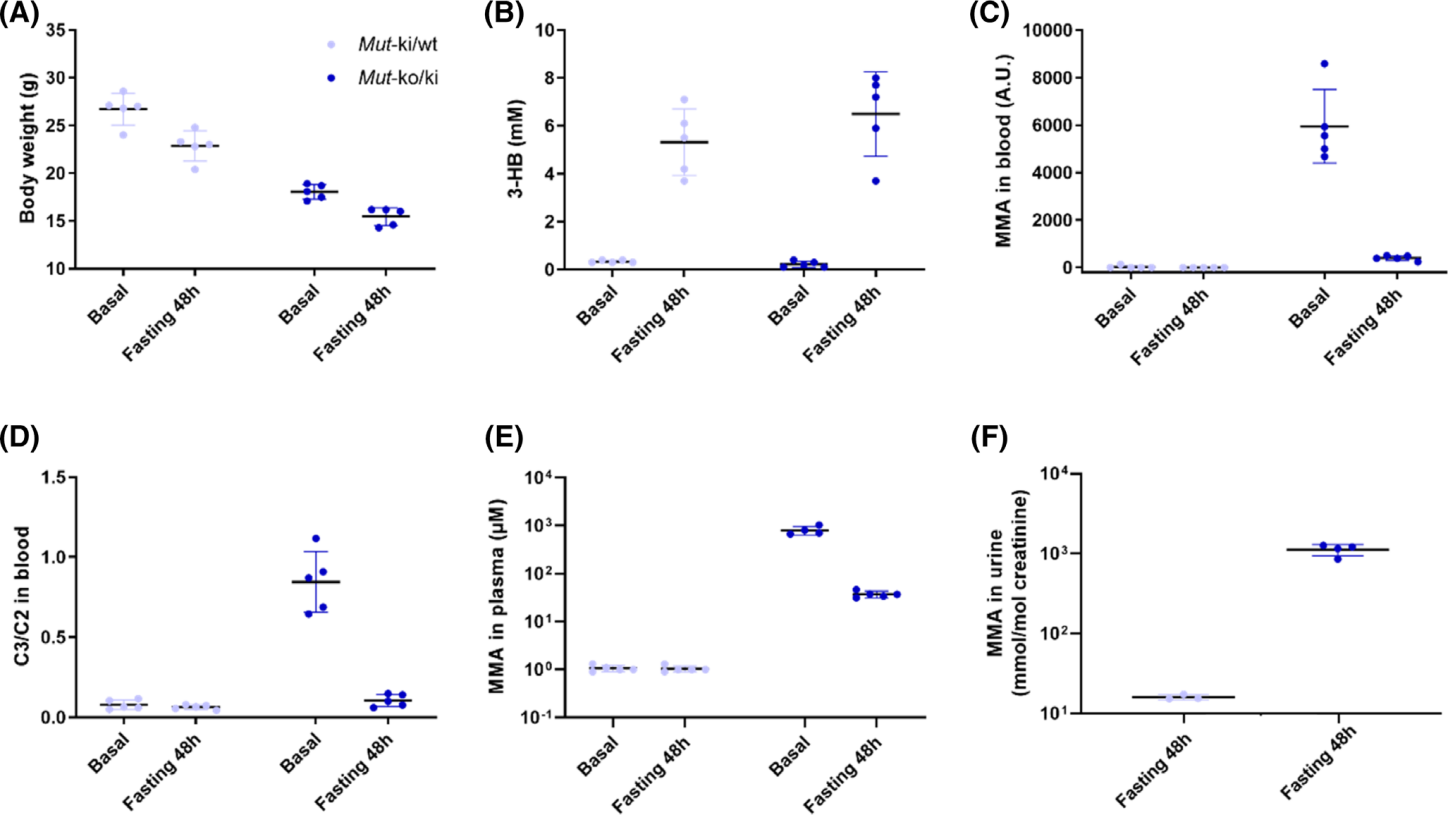
Reduction of metabolite levels upon 48‐hour fasting in mice fed a 51% protein diet. A, Changes in body weight, B, 3‐hydroxybutyrate (3‐HB), C, MMA and D, C3/C2 in dried blood spots were measured before and at the end of 48 hours of fasting in *Mut*‐ki/wt (n = 5) and *Mut*‐ko/ki (n = 5) females. E, MMA in plasma was measured in ad libitum fed or after 48 hours of fasting in cohorts of four to five mice. For Mut‐ko/ki mice: basal levels were 671 to 1030 μM, and reduced to 32 to 47 μM following fasting. F, For measurement in urine: samples from the same genotypes were pooled when insufficient volume. Fasted mice had MMA of 851 to 1260 mmol/mol creatinine, compared to 4540 to 97 520 mmol/mol creatinine when not fasted.^11^ In both *Mut*‐ko/ki and *Mut*‐ki/wt mice, body weight decrease (*P* < .001) and 3‐HB levels increase (*P* < .01) under fasting. In *Mut*‐ko/ki mice, MMA and C3/C2 levels in blood decrease (*P* < .01 and *P* < .001, respectively). Paired *t*‐tests were used for these analyses. MMA in plasma decrease in *Mut*‐ko/ki mice (*P* < .001, unpaired *t*‐test). Mice were 6 months of age. Baseline levels were determined 7 to 8 days before the food withdrawal

In order to investigate the role of dietary protein in this response, *Mut*‐ko/ki and *Mut*‐ki/wt females fed with a 51% protein diet from day 12 of life (a regular chow contains ~18.5% of protein), were fasted for 48 hours. Once again mice showed signs of fasting, including temporary weight loss (Figure 3A) and elevated blood ketone bodies (Figure 3B). *Mut*‐ko/ki mice also showed massively reduced MMA (Figure 3C) and C3/C2 (Figure 3D) in blood, as well as MMA in plasma (Figure 3E). Indeed, following 2 days of fasting, *Mut*‐ko/ki mice demonstrated MMA levels that were ~ 15 times lower in dried blood spots and ~ 20 times lower in plasma, and C3/C2 levels that were ~ 8 times lower. We further measured MMA in urine following fasting (Figure 3F). While after fasting urinary MMA was still elevated in mutants, comparison from a previous cohort on the same high‐protein diet without fasting,^11^ suggests that fasting resulted in a reduction of urinary MMA of approximately 40‐fold in *Mut*‐ko/ki animals. Creatinine levels were higher in fasted mice (*Mut*‐ko/ki: 4.2 ± 1.4 mmol/L; *Mut*‐ki/wt: 5.3 ± 1.4 mmol/L) than those in ad libitum fed conditions (*Mut*‐ko/ki: 1.7 ± 0.5 mmol/L; *Mut*‐ki/wt: 1.5 ± 0.8 mmol/L) for both mutants and controls, consistent with previous findings,^13^ but this increase of ~3‐fold does not compensate for the massive reduction of metabolite levels.

**FIGURE 4:**
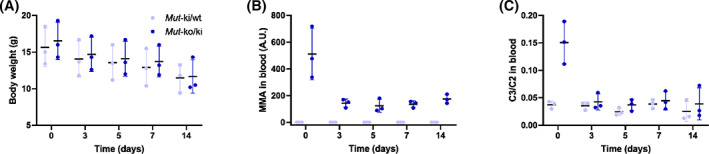
*Mut*‐ko/ki mice show reduced metabolite levels after switching to a 0% protein diet. A, Changes in body weight as well as, B, MMA and C, C3/C2 in dried blood spots were measured before and after switching from regular chow to a 0% protein diet in *Mut*‐ki/wt (n = 3) and *Mut*‐ko/ki (n = 3) females. Diet change was initiated at day 40 of life, indicated as Time 0. The first time point corresponds to the baseline level under a regular chow. Weight loss is not significant (*P* < .05); MMA and C3/C2 changes are significant by at least *P* < .001 between day 40 and all other days for *Mut*‐ko/ki. Two‐way analysis of variance with Tukey's multiple comparison tests were used for these analyses. Mice were switched to a 0% protein diet at day 40

Finally, to determine if the lack of dietary protein, which would be expected to be catabolized through the MMUT enzymatic pathway, was responsible for the reduced metabolite levels after fasting, we switched mice from regular chow to a 0% protein diet. Consistent with the fasting results, *Mut*‐ko/ki mice switched to a 0% protein diet showed slow weight loss (Figure 4A), as well as strongly reduced levels of MMA (Figure 4B) and C3/C2 (Figure 4C).

## 4 DISCUSSION

We examined the influence of fasting on the concentration of disease related metabolites in our mouse model of MMAuria. In contrast to our expectations, detectable levels of both MMA and C3/C2 reliably decreased following 24 and 48 hours of fasting. Although we did not perform any rigorous behavioral tests on these mice, cursory inspection also suggested there was no change in their behavior or condition (eg, movement disorders, gait abnormalities, or tremor). All of this is in apparent contradiction to current human treatment approaches, which stress the importance of keeping patients in an anabolic state in order to reduce the catabolic flux through the deficient propionyl‐CoA pathway.^3^, ^4^, ^14^, ^15^

There are a few potential explanations for the reduction of metabolite levels detected during fasting. The first is that the mice did not enter protein catabolism throughout the fasting period. We can be quite certain the mice were successfully fasted, as the time of fasting was relatively long (24‐48 hours), and they lost weight and had elevated circulating ketones consistent with what is expected for mice fasted for this period of time.^16^ In terms of protein catabolism specifically, historical research suggests that rats and mice lose 25% to 40% of their liver protein during 48 hours of starvation.^17^ More recent studies have noted a modest^18^ or no^19^ increase in blood urea following 16 to 24 hour fasting, suggesting a lack of protein breakdown at this time point. We did not measure plasma urea directly in this study. However, since in our model female mutant mice already have elevated plasma urea levels in ad libitum fed conditions,^11^ it is unclear if this would have been a useful marker. The avoidance of protein catabolism may be consistent with the results of switching mice from a normal or high‐protein diet to either a 0% protein diet or the fasted state, whereby in both conditions a similar drop in metabolites was found.

A second possibility is that the mice rid themselves of the extra metabolites through the urine. Analysis of urinary MMA in this study, compared to that of mice fed a high protein (51%) diet ad libitum in our previous study,^11^ suggests a strongly reduced MMA concentration in the urine in the fasted state. This data is in accordance with the reduced MMA concentrations in the plasma and dried blood spots; suggesting MMA in the blood and urine decrease correspondingly. MMA levels were always normalized to creatinine, which is often measured as a marker of urinary concentration.^20^ We found similar creatinine concentrations between mutant and control mice, suggesting a similar urinary volume. Although creatinine levels were higher in the fasted compared to the ad libitum fed condition for both mutant and control mice, this did not compensate for the massive reduction in MMA identified in the fasted mice. Therefore, it is unlikely that this reduced urinary MMA concentration is compensated by a massive increase in urinary volume.

A third potential explanation is that although fasting does induce catabolism, there is reduced flux through the missing methylmalonyl‐CoA mutase catalyzed step, and hence reduced metabolite production. This may come about either because there is avoidance of catabolism of propionyl‐CoA precursors (methionine, threonine, valine, isoleucine, odd‐chain fatty acids, side‐chain of cholesterol), or because an alternative pathway is used to catabolize propionyl‐CoA which by‐passes MMUT. To the former possibility, Manoli et al noted a hepatic gene expression profile consistent with a switch from fatty acid synthesis to gluconeogenesis upon fasting in their MMAuria mouse model.^21^ This was associated with a significantly altered expression of *Fgf21*, a peptide hormone involved in regulation of energy homeostasis and with a role in fatty acid oxidation regulation, including in the fasted state.^22^ These results suggest mice may try to use alternative energy production pathways to avoid metabolism of propionyl‐CoA precursors. To the latter possibility, a by‐pass pathway has been identified which sequentially utilizes the actions of *ACADSB*, *ECHS1*, *HIBCH*, *ADHFE1*, and *ALDH6A1* (human gene names) to create acetyl‐CoA from propionyl‐CoA, which is upregulated in *C. elegans* and HepG2 cells when they are vitamin B_12_ deficient or under genetic conditions mimicking MMAuria,^23^ and is active in rats.^24^ Both of the above possibilities are intriguing and warrant further investigation.

A study conducted in the 1990's on MMAuria and propionic aciduria patients in fasting conditions showed that protein catabolism accounted for ~52% of total propionate production, the rest originating from gut bacteria (~22%) and odd‐chain fatty acid oxidation during fasting (~30%).^7^ This was supported by parallel investigations which suggested the induction of odd‐chain fatty acid oxidation upon fasting, leading to the production of propionate independently from protein catabolism.^5^, ^6^ This is in line with the increased urinary excretion of propionate metabolites observed in propionic acidemia and MMAuria patients following 10 to 18 hours of fasting.^6^ Our data again appear to contradict these observations, indicating that further examinations are required to assess which pathways serve as the main sources of energy in both the fed and fasted states in the disease condition, in mice and humans. Furthermore, our findings open the question as to whether fasting is the direct cause of disease exacerbation in case of infection by opportunistic organisms. It may be worth further exploring the role played by the activation of innate immune signaling pathways as a trigger of metabolic crisis.

In conclusion, we identified fasting to result in reduced disease related metabolites in a mouse model of MMAuria, and suggest that the underlying basis for this and its potential impact on patient treatment should be further explored.

## AUTHOR CONTRIBUTIONS

Marie Lucienne designed and performed the experimental studies and contributed to the interpretation of the data and the writing of the manuscript. Déborah Mathis contributed to metabolite measurements in urine and plasma. Nathan Perkins and Ralph Fingerhut contributed to metabolite measurements in dried blood spots. Matthias R. Baumgartner conceived the idea for the project and contributed to the interpretation of the data. D. Sean Froese conceived the idea for the project and contributed to the interpretation of the data and the writing of the manuscript.

## CONFLICT OF INTEREST

All authors declare that they have no conflicts of interest.
